# Lycopodium Clinical Cases

**Published:** 1882-11

**Authors:** C. W. Butler

**Affiliations:** Montclair, N. J.


### LYCOPODIUM CLINICAL CASES.

C. W. Butler, M. D., Montclair, N. J.

Case I.—In the spring of '81 Mr. M. consulted me in regard to his wife, aged 39, who had suffered for eighteen years from the effects of uterine displacement due to maltreatment by a midwife in her first confinement. For seven years she had been under treatment by a prominent "regular" for constipation and other troubles, but in all that time found no relief except from opium and purgatives.

Her husband reported that the bowels were moved once in four to ten days by the use of "pills," and then with great pain. Colicy, cutting pains from right to left across the abdomen, worse after eating and in the afternoon, great bloating and sense of fermentation. Eats scarcely anything but crackers and tea, cannot touch meat of any kind. Menses excessively painful.

Prescribed, without seeing her, Lyc. 40 m. In two weeks reported that the bowels moved regularly once a day without pain, could eat anything, no colic or bloating and very little dysmenorrhœa, which improvement has continued up to the present time.

Case II.—J. M., female, age 11, very nervous temperament. Called in February 18th, 10 p. m. Face, flushed; pulse, 154; skin, hot and dry; restless; headache; great aching in back and limbs; no soreness of the throat or pain except on drinking cold liquids *other than water*. On examination found the whole throat much congested and four or five grayish spots the size of hemp-seed on the right tonsil. Lyc. 6x every two hours.

February 19th, 7 a. m. Right tonsil about the same as last night, except spots are smaller, but the left has a patch the size of a ten-cent piece, very angry looking, and a strong diphthetic odor in the room; pulse, 120; child very dull. Other symptoms the same. Lyc. 40 m. every six hours.

8 P. M. Throat much better, spots on the right side just visible, on the left the patch reduced to the size of a pea, the child quite bright.

20th, 9 A. M. Child up and apparently well; throat perfectly free from deposit or congestion; appetite good; no after symptoms.

CASE III.—L. B., female, age 9. Saw her for the first time, February 27th, 10 P. M. Just recovered from scarlatina; pulse too rapid to be counted; great aching of head, back and limbs; vomiting and flatus; throat pains on drinking cold milk, but *not cold water;* better from warm drinks; deposit grayish yellow covering the whole of the right tonsil; child very dull and difficult to rouse. Lyc. 40,m. one dose.

February 28th, 12 M. Child is playing about the room and taking care of the baby; throat clear; complains of nothing.

The indications on which Lyc. was selected in these cases were: Exudation *beginning* on the right side; stitches on swallowing; worse from *cold* drinks except water; better from *warm.* Aching pains in the back and limbs and the other Lyc. symptoms.

I would call especial attention to the aggravation from *cold* drinks other than water and amelioration from *warm* as being just contrary to what is given in most of the books. I have the fact noted on the margin of my *Hering* from some forgotten source (I think *Lippe's Mat. Med.*, but cannot verify the recollection) and have confirmed it again and again in the epidemic of the past three years in this locality.

Another point I may be permitted to note, is the immense superiority of the high or highest potencies in diphtheria, and also the advantage of giving the remedy time to act. The aggravation on the second day in Case I was due no doubt to the remedy and would not have occurred had a high potency been given and allowed to act. I am convinced that any one who will try the effect in diphtheria of high potencies in the single dose, prescribed in *strict* conformity to the law, will have no cause to repent the experiment or resort to local applications.

---

